# Subtelomeric study of 132 patients with mental retardation reveals 9 chromosomal anomalies and contributes to the delineation of submicroscopic deletions of 1pter, 2qter, 4pter, 5qter and 9qter

**DOI:** 10.1186/1471-2350-6-21

**Published:** 2005-05-17

**Authors:** Marie Sogaard, Zeynep Tümer, Helle Hjalgrim, Johanne Hahnemann, Birgitte Friis, Paal Ledaal, Vibeke Faurholt Pedersen, Peter Baekgaard, Niels Tommerup, Sultan Cingöz, Morten Duno, Karen Brondum-Nielsen

**Affiliations:** 1 The John F. Kennedy Institute, Glostrup, Denmark; 2 Wilhelm Johannsen Centre for Functional Genome Research, IMBG, The Panum Institute, University of Copenhagen, Denmark; 3 Department of Paediatrics, Glostrup Hospital, Denmark; 4 Department of Paediatrics, Roskilde Hospital, Denmark; 5 Department of Paediatrics, Sonderborg Hospital, Denmark; 6 Department of Medical Biology and Genetics, Dokuz Eylul University, Faculty of Medicine, Izmir, Turkey; 7 Department of Clinical Genetics, Rigshospitalet, Denmark

## Abstract

**Background:**

Cryptic chromosome imbalances are increasingly acknowledged as a cause for mental retardation and learning disability. New phenotypes associated with specific rearrangements are also being recognized. Techniques for screening for subtelomeric rearrangements are commercially available, allowing the implementation in a diagnostic service laboratory. We report the diagnostic yield in a series of 132 subjects with mental retardation, and the associated clinical phenotypes.

**Methods:**

We applied commercially available subtelomeric fluorescence in situ hybridization (FISH). All patients referred for subtelomeric screening in a 5-year period were reviewed and abnormal cases were further characterized clinically and if possible molecularly.

**Results:**

We identified nine chromosomal rearrangements (two of which were in sisters) corresponding to a diagnostic yield of approx. 7%. All had dysmorphic features. Five had imbalances leading to recognizable phenotypes.

**Conclusion:**

Subtelomeric screening is a useful adjunct to conventional cytogenetic analyses, and should be considered in mentally retarded subjects with dysmorphic features and unknown cause.

## Background

Mental retardation (MR) is a common disorder affecting 1–3% of the population, and yet the pathogenesis is only partly understood. Specific etiological factors are found only in about half of the patients, despite thorough clinical and laboratory investigations [1]. It is reasonable to believe that genetic factors are involved in many of the undiagnosed cases, since there is a generally increased recurrence risk for siblings [2], and a large number of different gene mutations are known to be associated with mental retardation. At present, using mental retardation as search word, at least 1000 items will turn out to be associated with mental retardation in the Online Mendelian Inheritance in Man (OMIM). In cases with prenatal onset of symptoms, growth retardation, malformations and dysmorphic features a chromosomal imbalance may play a role. The most common demonstrable genetic causes of global development delay involve chromosomal imbalance, the fragile X syndrome and Rett syndrome [3]. The available data indicate that chromosome abnormalities are found in 4–28% of individuals with mental retardation, and that severity of MR and the presence of congenital anomalies increase the diagnostic yield of chromosome abnormalities [4].

The subtelomeric regions are gene-rich and are often involved in chromosomal rearrangements [5]. Since 1995 it has been recognized that subtle rearrangements at the telomere regions may account for a proportion of cases with unexplained mental retardation [1]. A number of techniques can be applied for subtelomeric screening, for instance FISH with subtelomeric probes, analysis with microsatellite markers or high resolution comparative genome hybridization (HR-CGH) [6]. Recently, the use of MLPA (multiplex ligation dependent probe amplification) [7], and microarrays [8] were described. A recent review of over 2500 tested and reported subjects with mental retardation revealed the presence of subtelomeric rearrangements in approximately 5 % of the cases. [9].

In this study we have investigated 132 mentally retarded patients for subtelomeric rearrangements by FISH analysis using a commercially available system (Cytocell ^R^). We found 9 rearrangements (two were siblings) among 113 cases with both MR and dysmorphic features. Three of the rearrangements were characterized previously by molecular means [10], and in this study we have delineated the size of the deletion in one case with a 2q telomere abnormality.

## Methods

### Patients

A total of 132 cases from 131 families were referred to the John F. Kennedy Institute for subtelomeric screening. They were all examined in a period between January 1998 and February 2003. A previous cytogenetic analysis at the 500–550 band stage was normal in 130 cases, and in two cases an unresolved subtle chromosome anomaly was suspected. Of these patients 113 cases had mental retardation together with dysmorphic features. Sixteen of these cases had a positive family history of MR and dysmorphic features. In 19 patients mental retardation was present without apparent dysmorphic features. Four of these cases had a positive family history of MR. In all cases where a subtelomeric abnormality was detected the parents were investigated if available.

### Subtelomeric screening, FISH analysis, and quantitative PCR

Subtelomeric regions were screened with the Chromoprobe Multiprobe T system (Cytocell). The analysis was performed as instructed by he manufacturer. With this system a simultaneous analysis of 41 chromosome arms, except for the p arms of the acrocentric chromosomes is possible. The signals were detected and analyzed in a fluorescence microscope. All abnormal or ambiguous results were reanalyzed using a specific subtelomeric probe for the suggested abnormality (Cytocell aqua or TelVysion (Vysis)).

To establish the size of the deletion in patient 3, FISH analyses were carried out using 19 BAC clones from the RP11 library mapping to 2q37.2-q37.3 (Human Genome Browser). FISH analysis was carried out using 200 ng BAC DNA using standard protocols. Probe DNA was labeled with biotin-11-dUTP (Boehringer Mannheim) and signals were visualized using avidin-FITC detection system.

To establish if the deletion in case 8 included the *NSD1 *gene real-time quantitative PCR was carried out: A reference PCR amplifying a 51 bp genomic fragment of the *GAPDH *gene and a test PCR allowing an amplification of a segment in exon 5 of *NSD1*, was developed according to the Primer Express/SDS7000 guidelines and evaluated on a SDS7000 real-time PCR utility (Applied Biosystems). DNA from patient 8 and two controls were isolated and diluted (approx 5 ng/ml) to obtain nearly exact cycle threshold values (C_T_) for the reference PCR. The comparative C_T _method was used to calculate the relative quantitative relation between the two PCR reactions, and showed a steady 1:0.5 relation in the patient sample (consistent with a deletion of the *NSD1 *gene)

### Case Reports

#### Patient 1

**Figure 1:**
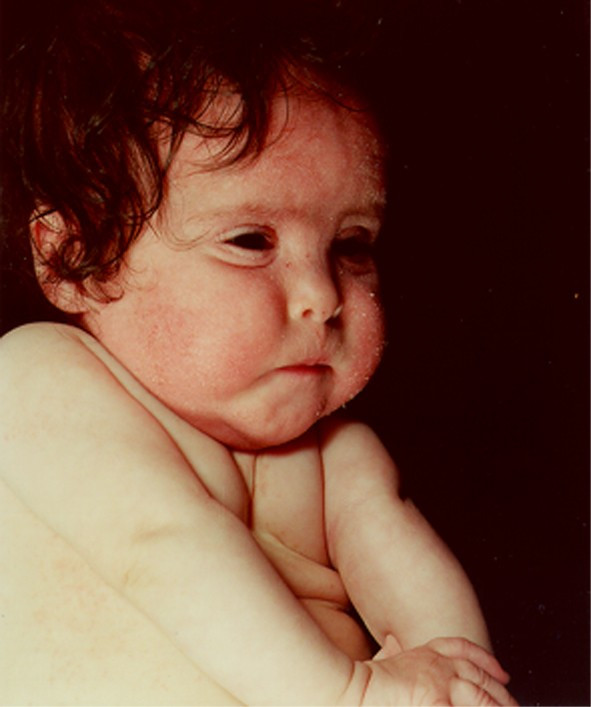
Facial features of patient 1 with 1p deletion (1 year old).

Patient 1 was a female born to unrelated healthy parents at 40 weeks of gestation. She had a healthy older brother. Birth weight was 2600 g, birth length 49 cm, and head circumference was 31 cm. Apgar scores were 7/1, 9/5, 10/10. The patient showed prenatal- as well as postnatal growth retardation (-3/-3 1/2 SD). She was hypotonic and severely delayed in psychomotor functions. The patient died of pneumonia 3 years old. At that time she was only able to sit with support and had no language except from babbling. She had dysmorphic features including prominent forehead, high-arched palate, flat mid-face with small nose, small palpebral fissures, long philtrum, small mouth with thin lower lip, small hands and feet and vision abnormalities in the form of markedly delayed visual maturation. In addition she had severe skin problems with suppurate eczema in periods. No other cases of mental retardation were known in the family. Clinical features are shown in fig. 1. An autopsy did not reveal any organ malformations.

Her karyotype was 46,XX.ish del(1)(p36.3). Her parents had normal karyotypes without subtelomeric rearrangements. Hence, her deletion at 1p36 was *de novo*. The size of deletion was characterized at the molecular level in a previous study [10], comprising 8 Mb.

#### Patient 2

The boy was born as the first child to unrelated healthy parents. The father had two apparently healthy children from a previous relationship. During pregnancy the fetus showed growth retardation. He was born at 39 weeks of gestation with a birth weight of 2125 g. Apgar scores were 8/1, 10/5. He was born with anal atresia, subsequently operated with good results. He had MR and facial dysmorphic features. He showed growth retardation and was found to have a mild delay of motor function at 21 months. He could walk at the age of 2 1/2 years but had no spoken language; instead he used sign language. He had congenital hearing- and vision-impairment with hypermetropia, which were corrected with hearing aid and glasses respectively. Ophthalmological examination revealed optic atrophy. No other cases of mental retardation were known in the family. Cytogenetic analysis revealed additional material on the long arm of chromosome 2. Whole chromosome painting with a chromosome 2 probe did not paint the additional material Subtelomeric screening showed subtelomeric signals on both chromosomes 2 and furthermore, a signal from the subtelomeric probe from chromosome 22q was localized to the additional material on chromosome 2 demonstrating that the extra material derived from 22qter. (Normal subtelomeric signals were also present on 22q). The mother had a normal karyotype without subtelomeric rearrangements, and the father was not available for examination. The karyotype was 46,XY.ish der(2)t(2;22)(q37.2;q1?). Hence, the patient had an unbalanced karyotype with a partial trisomy for the long arm of chromosome 22.

#### Patient 3

The patient was born as number 2 of 2. She was delivered by Cesarean section at 41 weeks of gestation because of her size and previous section. Birth weight was 5500 g and birth length 58 cm. She had MR with mild delay of motor function and walked alone 16 months old. She had recurrent airway infections and bronchitis. Growth was normal, but her head was large (+3SD). MRI of the brain was normal. She had recurrent fractures and she was diagnosed with osteoporosis using DEXA scanning 6 years old. Her height was 119 cm and weight 25 kg at age 7 years. She had normal levels of calcium, phosphate, magnesium and basic phosphatase. Her thyroid status was normal. Dysmorphic features included frontal bossing, narrow and low dorsum of nose and hypertelorism. No other cases of MR were known in the family.

Her karyotype by subtelomeric screening was 46,XX.ish del(2)(q37.2). Her mother showed the same subtelomeric abnormality which is a known polymorphism of D2S9886. However, further FISH analyses were carried out as described.

#### Patient 4

**Figure 2:**
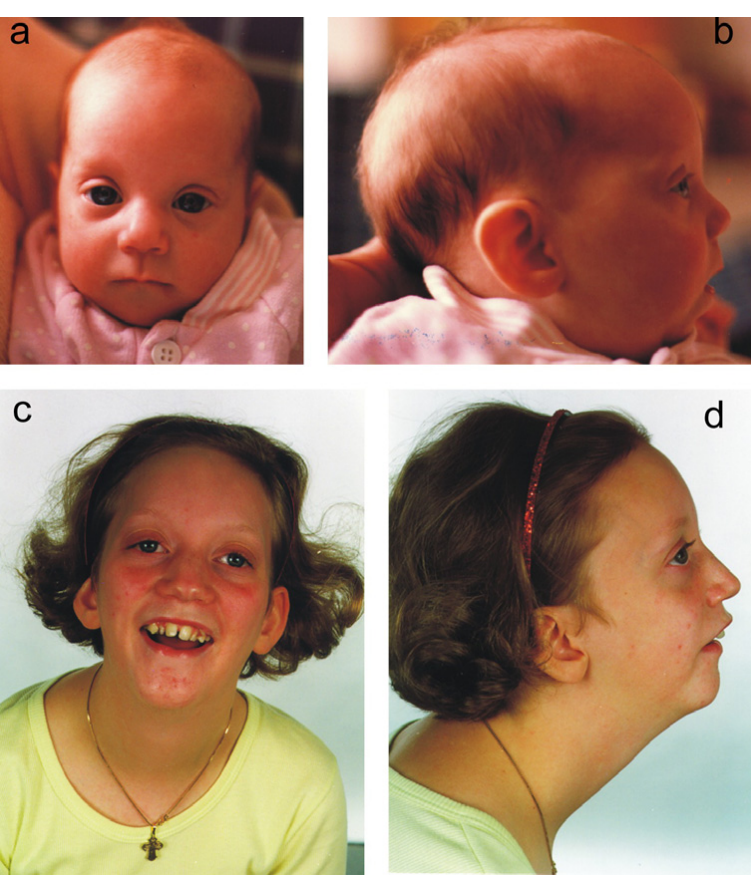
a,b,c,d. Patient 4 aged 3 months (a,b) and 13 years (c,d) with 4p deletion.

The patient was born to healthy, unrelated parents. She was born at 40 weeks of gestation with a birth weight of 2500 g, birth length of 46 cm and head circumference of 28.6 cm. Apgar scores were 10/1, 10/5. One older sibling was healthy. She thrived poorly and was found to be generally hypotonic 2 months old. Her development was retarded and she was admitted to hospital several times with infections and febrile convulsions. A chromosome analysis and metabolic screening at the time was normal. She was treated for epilepsy from the age of 2 years. Feeding problems were pronounced, and she was considered to have an autistic disorder. Her development corresponded to approximately 11–15 months at the age of 2 1/2 years. As she grew up she became fond of eating. MRI of the brain at the age of 9 years was normal. Menarche occurred at the age of 11 years. She had well-developed gross motor and some language skills at the age of 15 years. She was referred to subtelomere chromosome analysis at the age of 12 years because of MR, dwarfism with growth corresponding to -3SD (146 cm and 43 kg), hypotonia and dysmorphic features including microcephalia, micrognathia and protrusion of the eyeballs. See figure 2 for clinical features.

Her karyotype by subtelomeric screening was 46,XX.ish del(4)(p16.1). The parents had normal karyotypes without subtelomeric rearrangements. Hence, she had an unbalanced karyotype with monosomy 4p *de novo*.

#### Patient 5

This patient was 27 years old at the time of reinvestigation. Pregnancy, birth and neonatal period were reported as normal. At age 3 years he was referred for chromosome analysis because of mild MR and delay of motor function. Slight dysmorphic features (round facies, small head) were noted. The chromosome analysis (performed both in peripheral blood lymphocytes and cultured skin fibroblasts) revealed additional material on the short arm of chromosome 22 in about 25 % of the metaphases analyzed. At that time it was not possible to reveal the origin of this extra material. At age 27 years he was mildly mentally retarded and worked in a sheltered workshop. His height and weight were normal. Subtelomeric screening showed a signal from the subtelomeric probe12p (in addition to the normal signals on chromosome 12p) localized to the short arm of chromosome 22. His karyotype by subtelomeric screening was 46,XY.ish der(22)t(12;22)(p13;p?). Furthermore, Multicolor FISH confirmed the presence of chromosome 12 material on 22p in approx. 25% of the metaphases. Hence, he had an unbalanced mosaic karyotype with a partial trisomy for chromosome12pter.

#### Patient 6a, b (and c)

**Figure 3:**
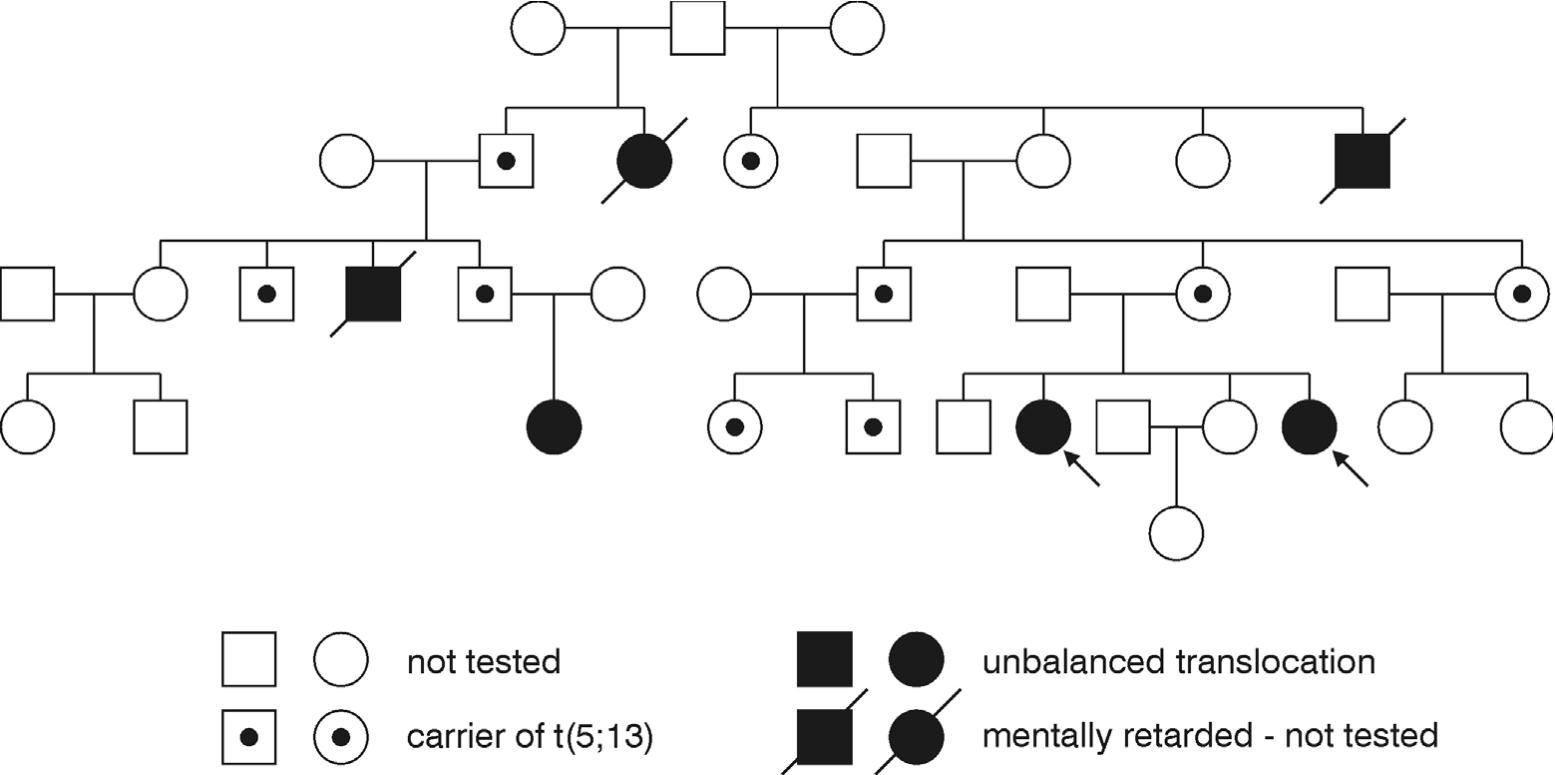
Pedigree for patients 6a & 6b & 6c illustrating segregation of t(5;13) translocation. Filled symbols are mentally retarded individuals, dotted symbols are carriers of the translocation. Arrows point to the two probands.

The patients are two sisters (6a,b), who are distantly related to a girl with a similar phenotype (6c) (figure 3). The two sisters were born in 1966 (6a) and 1976 (6b) to healthy, unrelated parents. Patient 6a was born at term with a birth weight of 2900 g and birth length of 48 cm after an uncomplicated pregnancy. Apgar scores were unknown, but amnion fluid was green and the baby was placed in an incubator. Feeding was poor in the neonatal period. One year old her weight was 8700 g and the height was 75 cm. Today (37 years old) her weight is 48 kg and the height is 154 cm. She has scoliosis, small head circumference and spastic gait. She has severe MR and needs help with almost everything. The fine and gross motor skills are poor. (According to the parents she likes being with other people and is generally a happy person).

The younger sister (6b) was also born at term after normal pregnancy and delivery. The birth weight was 3470 g and birth length 48 cm. She had congenital dislocation of the hip and one week old a brace was applied. According to the parents she resembled her older sister, but was much stronger and was able to suckle by herself. One year old her weight was 7300 g and height was 73 cm. She was hyperactive for a number of years with a very little sleep demand. Today (27 years old) she weighs 44 kg and the height is 144 cm. She has severe MR and is dependent on constant assistance. Both sisters have good health in general.

The third girl (6c) within this family was born in 1996 as the first child to healthy parents. Birth weight 1935 g, length 44 cm, head circumference 29.6 cm. She has moderate/severe mental retardation without language. She is growth retarded with microcephalia (head circumference below -3 standard deviations)

Subtelomeric screening revealed an unbalanced translocation in the sisters, and a balanced translocation in their mother. The unbalanced karyotype was 46,XX.ish der(13)t(5;13)(q35.2;q34). Thus the patients had partial monosomy 13qter as well as partial trisomy 5qter. Case 6c had the same unbalanced karyotype.

#### Patient 7

Patient 7 was a girl born at 42 weeks gestation as the first child to healthy, unrelated parents. The mother previously had two spontaneous abortions. Birth weight was 2770 g, birth length 47 cm and head circumference 30.5 cm. She had a hemangioma on the forehead and a neonatal tooth. She was severely delayed in development with hypotonia and postnatal growth retardation and developed epilepsy. She had dysmorphic features including hypertelorism, narrow eye fissures, broad nasal bridge, large philtrum, abundant head- and body hair, clubfoot and atrial septal defect (ASD). She was severely retarded and died 3 years old from pneumonia.

Subtelomeric screening showed an unbalanced translocation inherited from a balanced translocation in the father. Her karyotype was 46,XX.ish der(9)t(9;22)(q34.2;q13.3)*pat*.

#### Patient 8

**Figure 4:**
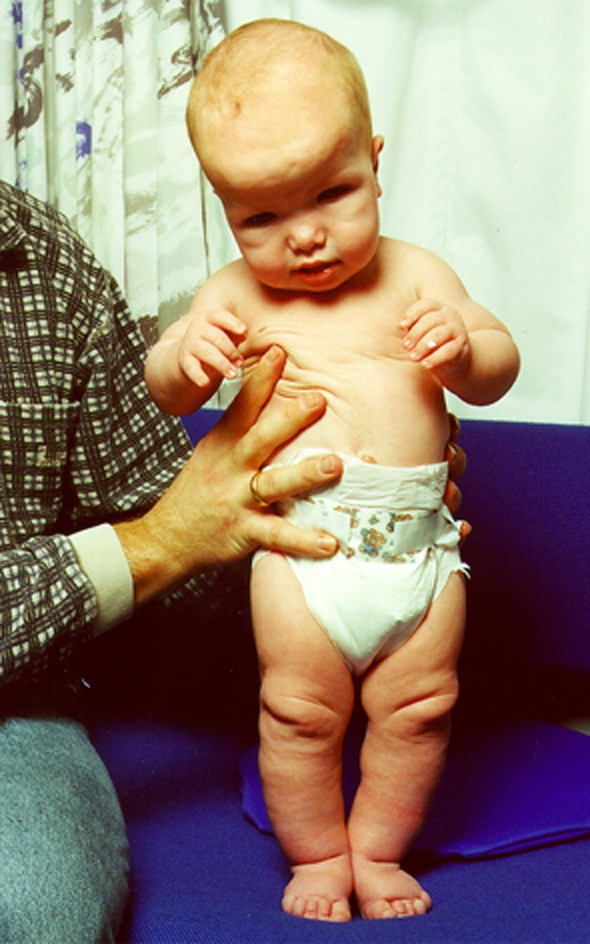
Patient 8 with Sotos syndrome (9 months old).

The patient was a girl born to healthy, unrelated parents. Two older maternal half-sibs were healthy. The pregnancy was complicated by polyhydramnios and she was delivered at 40 weeks of gestation by Cesarean section due to slow progress in labor. Forceps was used through the uterotomia because of macrocephaly. Birth weight was 3955 g, birth length 55 cm, and head circumference 40 cm. The girl was asphyctic with apgar scores 2/1, 7/3, 9/5, and 10/10. Edema of hands and feet were noticed at birth and the neonatal period was complicated by hypoglycaemia. An ultrasound scan of the brain at age 3 days showed signs of haemorrhage in the lateral ventricles, small periventricular cavities, and suggested corpus callosum hypoplasia. The latter was verified at MRI of the brain at 4 weeks of age and in addition enlarged lateral ventricles were present. At 11 months of age renewed MRI in addition showed partial agenesis of gyrus cinguli and periventricular leukomalacies. At 12 months of age significant dysmorphic features, as macrocephaly, dolicocephaly, frontal bossing, receding frontal hairline, deep-set eyes with epicanthus, nystagmus, depressed nasal bridge, and a protruding tongue were noticed. The trunk of her body was long compared to the extremities. Finger- and toenails were thin, brittle and deep set with periungual edema and a tendency to develop paronychion. Skin of the palms and foot soles were thickened, fingertip pads were prominent, and the thumbs were broad and adducted (fig. 4). She was hypotonic. Echocardiography was normal. X-ray examinations showed that metatarsal bones were short and metacarpal bones were short and broad. Furthermore, the bone age at 23 months was dissociated with phalangeal bone age corresponding well to chronological age, but carpal bone age corresponding to 6 months. Her motor and mental milestones as well as expressive language were delayed. Growth at 26 months of age showed: weight +1 SD, height +1,5 SD, and head circumference +3–4 SD. During the first two years she had recurrent urinary and upper respiratory tract infections. At 29 months age hypermobility of joints, redundant skin, a small umbilical hernia, mild kyphoscoliosis and contractures of knees were observed. Due to the phenotype the girl was suspected of having Sotos syndrome.

Subtelomeric screening revealed the karyotype 46,XX.ish del(5)(q35). Quantitative PCR revealed that the *NSD1 *gene was deleted, confirming the diagnosis of Sotos syndrome.

## Results

In this study we analyzed 132 patients (clinical features summarized in table I) by subtelomeric screening, where conventional chromosome analyses were normal in 130 cases. Table II summarizes the results. Seven cases (including two sisters) had cryptic aberrations not visible by conventional cytogenetic analysis. Both novel as well as previously described subtelomeric aberrations were identified. Furthermore, in two cases (patient 2 and patient 5) an unresolved visible small structural abnormality of chromosome 2 and 22, respectively, was suspected and resolved by subsequent subtelomere testing. In patient 2 the extra material was shown to be due to partial trisomy for the long arm of chromosome 22. Apparently no specific phenotype is associated with cases with this karyotype. However, anal atresia (observed in our patient) is present in patients with cat eye syndrome due to partial tetrasomy of the 22q11 region [11]. Patient 5 who was mildly retarded with few dysmorphic features was found to have a mosaic karyotype with partial trisomy 12p in about 25% of cells. More than two dozen patients with duplication of 12p have been described, mostly with severe mental retardation. Pallister-Killian syndrome is associated with tetrasomy (12p) mosaicism in fibroblasts [11]. Our patient did not resemble these phenotypes.

Patients 6a, 6b and 6c had both inherited the same submicroscopic chromosomal imbalance, I e partial monosomy 13qter and partial trisomy 5qter, due to an unbalanced translocation which segregated in balanced form through several generations in the family. The 3 affected relatives were severely retarded with microcephaly.

For patient 3 the extent of the deletion at 2q37 was delineated further with detailed FISH, prompted by the finding that the normal mother had apparently the same deletion with the probe set from Cytocell(R) which is recognized as a wellknown polymorphism [12]. In the child the most distal BAC clone, which was present on the abnormal chromosome 2 was RP11-1006P17, while the distal overlapping BAC clone RP11-473L20 was deleted. The distal breakpoint of the deletion was thus mapped within a 100 kb region (Human Genome Browser chromosome position 236,195,870-236,292,756) at cytogenetic band 2q37.2 and the deletion extended further to the telomere. The size of the deletion was thus approximately 6.8 Mb. Monosomy of 2q37 has been reported in more than 60 patients and recently deletion mapping of 20 cases has been published [13]. Monosomy 2q37 patients show significant clinical variability mainly dependent of the size of the deletion, though some degree of mental retardation and facial dysmorphism have been recognized in all patients, as it is also valid for the present case. In the present case the deletion breakpoint is mapped within or at the promoter region of *CENTG2 *gene between the microsatellite markers D2S336 and D2S338, which have been used in the study described by Aldred et al. [13]. In this study a critical interval for brachymetaphalangism, which is the main symptom of Albright hereditary osteodystrophy (AHO)-like brachymetaphalangism, has been assigned to the 3 Mb region from *HDAC4 *gene to the telomere. This region is deleted in the present patient, who does not present this feature. Brachymetaphalangism was suggested to be partially penetrant and some patients deleted for this region show other severe skeletal abnormalities. Our patient suffers osteoporosis, which also might be due to the mutations of the same gene leading to different degrees of symptoms. Recently Giardino et al. [14] published a patient with AHO-like syndrome where the deletion breakpoint of the patient was within BAC clone RP11-585E12, approximately 1.6–18 Mb distal to the breakpoint of the present case. This region includes five genes (*CENTG2*; *GBX2*; *ASB18*; *CMKOR1*; *FLJ22527*), which are deleted from the present case but not from the case described by Giardino et al. [14]. However at present it is difficult to predict the effects of the protein products on the different phenotypes observed in these patients. The phenotypically normal mother only had a deletion with probe D2S2986, but was not deleted with other probes. .

## Discussion

Several conclusions can be drawn from the present study:

In 113 patients with both mental retardation and dysmorphic features we have identified subtelomeric abnormalities in 9 patients, and this result corresponds well with other studies. In a recent review of 20 studies the mean detection rate was found to be 4.8%, ranging between 0%-23% [9]. In our study 19 subjects with apparently non-syndromic idiopathic mental retardation, with or without positive family history were investigated. None of these patients showed rearrangements. This is in accordance with the study of Joyce et al. [15] who showed that cryptic telomeric rearrangements were not a significant cause of idiopathic mental retardation. It can be speculated that especially in familial cases of idiopathic mental retardation mendelian or multifactorial inheritance is more often the cause.

Our series represent referrals to a diagnostic laboratory for various reasons but undiagnosed mental retardation with dysmorphic features suggesting a "chromosomal phenotype" account for the majority. It is reasonable to assume that different detection rates are due to selection criteria, and it is likely that stringent selection criteria like the 5-item checklist (family history of MR, prenatal onset growth retardation, postnatal growth abnormalities, facial dysmorphic features, non-facial dysmorphism and/or congenital abnormalities) provided by de Vries et al. [16] will increase the detection yield of chromosome abnormalities. This can also be seen in the study reported by Walter et al. [17], where the authors have performed a subtelomere FISH study of 50 unrelated children ascertained by a checklist that evaluates MR or development delay, dysmorphism, growth defect, and abnormal pedigree and found ten causal rearrangements (detection rate of 20%).

The second conclusion is that some abnormalities are recurrent and not very rare.

For most of the cases a genotype-phenotype correlation was present. Patient 1, with deletion at 1pter had a phenotype correlating well with the 1p- syndrome, a now well-described syndrome and probably the most common terminal deletion syndrome [18-20]. However, it should be kept in mind that phenotype of these cases are partly dependent on the deletion size. The same conclusion can also be drawn for patient 3 with deletion at 2qter.

Patient 4 had deletion of 4pter including the Wolf-Hirshhorn syndrome region. This patient's phenotype was relatively mild and correlated with some patients previously described with similar 4p16.3 microdeletions [21,22]. Some patients have been designated Pitt syndrome (Pitt-Rogers-Danks syndrome, PRDS), but recently it was argued that Pitt and Wolf-Hirshhorn syndromes represent phenotypic variations of the same microdeletion [23].

Patient 7 had the karyotype, 46,XX.ish der(9)t(9;22)(q34.2;q13.2q13.3)*pat*. This patient was monosomic for distal 9q and trisomic for distal 22qter. An emerging phenotype of patients with distal 9q-deletions has been suggested, characterized by mental retardation, hypotonia, microcephaly, synophrys, short nose with anteverted nares, midface hypoplasia, a tented upper lip with a large, protruding tongue and sometimes neonatal teeth. Congenital heart disease and seizures are common complications [24]. These features were overlapping with those of the present case. It has also been suggested that 9qter deletions can cause syndromic obesity in children [24,25], but this was not the case for patient 7. Patient 8 had deletion of 5qter, which was in line with the clinical suspicion of Sotos syndrome. The *NSD1 *gene defective in Sotos syndrome is mapped at 5q35, 4.2 Mb from the telomere. Real-time quantitative PCR analysis demonstrated subsequently that the deletion in the patient comprised exon 5 of *NSD1*, confirming the clinical diagnosis. The deletion in our patient encompasses the subtelomeric region, the *NSD1 *gene and potentially further chromosomal material towards the centromere. Nagai et al. [26] observed that patients with a deletion involving the entire *NSD1 *gene and several other genes exhibited abnormal features of the CNS, the cardiovascular and the urinary systems, while these features were absent in patients with *NSD1 *point mutations. In addition to the features of Sotos syndrome, our patient showed several features consistent with Weaver syndrome [27]. A clear clinical distinction between these two syndromes is however difficult, and it is still discussed whether they result from allelic heterogeneity or are indeed distinct syndromes [28,29]. Recently, *NSD1 *mutations have been found in some cases of Weaver syndrome patients arguing for allelic heterogeneity [30].

A third conclusion is that a significant proportion of subtelomeric abnormalities are due to familial translocations, which is in accordance with previous studies [31,32]. This is important for genetic counseling.

In summary our study confirms the diagnostic value of subtelomeric screening, especially in mentally retarded subjects with dysmorphic features. A family history is of additional significance. Finally our study can contribute to the delineation of syndromes like the 1p- syndrome, the 2q- syndrome, the 4p- syndrome, the 9q- syndrome and the 5q- Sotos syndrome.

## Competing interests

The author(s) declare that they have no competing interests.

## Authors' contributions

MS carried out genotype-phenotype analyses, participated in the design and coordination of the study and drafted the manuscript. ZT, SC and NT carried out the molecular analyses and FISH mapping of patient 3. JH supervised the subtelomeric and cytogenetic analyses. HH, BF, PL, VFP and PB contributed detailed clinical data and photos of patients. MD performed real-time quantitative PCR. KBN conceived of the study, and participated in its design and coordination. All authors read and approved the final manuscript.

## Pre-publication history

The pre-publication history for this paper can be accessed here:



## Figures and Tables

**Table 1:** Clinical features for the nine patients with rearrangements.

**Case**	**Dysmorphic facial features**	**Other dysmorphic features/ abnormalities**	**OFC (cm)**	**BW (g)**	**GA (weeks)**
**1**	Yes	Small hands and feet	31	2600	40
**2**	Yes	Anal atresia	-	2125	39
**3**	Yes	No	-	5500	41
**4**	Yes	Dwarfism	28.6	2500	40
**5**	Yes	No	-	-	-
**6 a**	Yes	Scoliosis	-	2900	40
**6 b**	Yes	Congenital dislocation of the hip	-	3470	40
**7**	Yes	Clubfoot, atrial septum defect, abundant head- and bodyhair	30.5	2770	42
**8**	Yes	Adducted and broad thumbs, thickened skin in sole of foots and palms	40	3995	40

OFC, occiput frontal circumference; BW, birth weight; GA, gestational age

**Table 2:** Results of subtelomeric FISH for the nine patients with rearrangements.

**Case**	**Family history of MR**	**Karyotype**	**Deletion/duplication size**	**Parents karyotype**
**1**	No	46,XX.ish del(1)(p36.3)	8 Mb (a)	Normal
**2**	No	46,XY.ish der(2)t(2;22)(q37.2;q1?)	NA	Mother normal / father NA
**3**	No	46,XX.ish del(2)(q37.2)	6.8 Mb	Mother normal (e) / father NA
**4**	No	46,XX.ish del(4)(p16.1)	Approx 4 Mb (d)	Normal
**5**	No	46,XY/46,XY.ish der(22)t(12;22)(p13;p?)	NA	Normal
**6a,b**	Yes	46,XX.ish der(13)t(5;13)(q35.2;q34)	3.9 Mb (13qter) and 6.5 Mb (5qter) (b)	Mother: 46,XX,t(5;13)(q35.2;q34)
**7**	Yes	46,XX.ish der(9)t(9;22)(q34.2;q13.3)nat	4.1 Mb (9qter) and 5.7 Mb (22qter) (c)	Father: 46,XY,t(9;22)(q34.2;q13.31)
**8**	No	46,XX.ish del(5)(q35)	4–7 Mb (f)	Normal

(a),(b),(c), Cases 15, 16, 14, respectively, described by Schoumans et al. (2004); (d) also deleted for Wolf-Hirshhorn probe (Vysis ^®^); (e), Deletion D2S2986 polymorphism (see text for detail); (f), see text for detail; MR, mental retardation; NA, not analyzed.
